# Involvement of *Escherichia coli-*encoded Lon protease and its substrates in phenotypic antibiotic resistance elicited by 4-amino-2-nitrophenol

**DOI:** 10.1128/aem.01603-25

**Published:** 2025-12-09

**Authors:** Santhi Sanil Nandini, Sirisha Jagdish, Subinoy Rana, Dipankar Nandi

**Affiliations:** 1 Department of Biochemistry, Indian Institute of Science 29120, Bengaluru, Karnataka, India; 2 Materials Research Centre, Indian Institute of Science 29120, Bengaluru, Karnataka, India; Danmarks Tekniske Universitet The Novo Nordisk Foundation, Kgs. Lyngby, Denmark

**Keywords:** 2,4-DNP, Lon protease, nitroreductases, bioremediation

## Abstract

In prokaryotes, the energy-dependent protein degradation is controlled, primarily, by two ATP-dependent proteases, Lon and Clp. This study investigates the roles of *Escherichia coli* (*E. coli*)-encoded Lon protease in the metabolism of 2,4-dinitrophenol (2,4-DNP), a toxic industrial compound. Enhanced conversion of yellow-colored 2,4-DNP to a reddish-brown product was observed in a strain lacking Lon protease (Δ*lon*). This observation led us to characterize and understand the mechanisms of 2,4-DNP metabolism. UV-visible and LC-MS analyses revealed differences in the conversion products between the wild-type and Δ*lon*. One of the substrates of Lon protease is MarA, a transcription factor, and studies with different mutants followed by *trans* complementation demonstrated MarA-dependent conversion. The bathochromic shift of spectral peaks suggested reduction processes and possible involvement of nitroreductase enzymes. Indeed, the expression of two genes encoding nitroreductases, *nfsA* and *nfsB*, increased with 2,4-DNP and was dependent on MarA. Importantly, the production of the reddish-brown product was lower in strains lacking *nfsA* or *nfsB*. Finally, LC-MS analysis identified one of the conversion products of 2,4-DNP to be 4-amino-2-nitrophenol (4,2-ANP). Dose studies with purified 4,2-ANP demonstrated that it did not lower the growth of *E. coli* (unlike 2,4-DNP) and induced phenotypic antibiotic resistance in an *acrB-*dependent (like 2,4-DNP) but in a *marA-*independent (unlike 2,4-DNP) manner. This study revealed how *E. coli* in the environment converts a toxic compound (2,4-DNP) into a lesser toxic compound (4,2-ANP) and helps survive in the presence of antibiotics. Overall, this study contributes to our understanding of biological responses to nitroaromatics.

**IMPORTANCE:**

*E. coli* is one of the common microorganisms in feces-contaminated sewage and often interacts with several pollutants. This study identifies the roles of Lon protease and its substrate MarA in inducing nitroreductases, NfsA and NfsB, in reducing toxic 2,4-DNP to less toxic 4,2-ANP, a novel inducer of phenotypic antibiotic resistance in *E. coli*. This study sheds light on the roles of *E. coli*-encoded Lon protease upon exposure to harmful nitroaromatics. Common environmental pollutants can act as a selective pressure, favoring the survival as well as proliferation of bacteria containing antibiotic-resistant genes, which can easily be transferred to other bacteria through horizontal gene transfer. This study offers insights into mitigation methods in *E. coli*, a well-characterized model. It is possible that such environmental pollution strategies may be translated to other models, such as *Pseudomonas*, which are commonly used in bioremediation studies.

## INTRODUCTION

Toxic compounds can be categorized into natural compounds, by-products of metabolism, xenobiotic compounds, products of human activities, etc. Due to their complex structures, xenobiotics often exhibit low biodegradability, posing significant environmental challenges. Since these compounds have a very recent origin, organisms that can degrade these compounds are limited (1). The compound 2,4-DNP is a di-nitro aromatic organic compound with the chemical formula HOC_6_H_3_(NO_2_)_2_. It is a refractory pollutant released into the environment from industrial effluents, spill-outs, or through the degradation of pesticides containing 2,4-DNP. According to the US Environmental Protection Agency, it is a priority pollutant whose concentration in natural water sources should be limited to 10 mg/L (2).

Several physical and chemical methods are known for treating nitrophenol pollutants, but they are not safe and cost-effective. Energy-intensive chemical treatments such as incineration may be too expensive at low concentrations or may cause other environmental problems such as NO_x_ emissions. Other relevant biological procedures that are safe and cost-effective are being investigated for the purpose. Some microbes have developed mechanisms to degrade toxic compounds through reductive pathways despite the structural rigidity conferred by the delocalization of electrons in aromatic rings (3, 4). However, safe and inexpensive strategies to reduce nitrophenolics are of concern and an important area of study.

Cellular proteolysis is used to remove aberrant and misfolded proteins and restrict the quantity and duration of essential regulatory proteins. In prokaryotes, energy-dependent protein degradation is controlled majorly by two classes of proteases: cytoplasmic and membrane-bound. The major cytoplasmic proteases are Lon (serine protease), Clp (serine protease), and HS1UV (threonine protease), whereas FtsH (metalloprotease), EcfE (zinc metalloprotease), and DegS (serine protease) are membrane-bound (5–9). The Lon protease is a member of the AAA^+^ family of proteins with three domains: amino-terminal domain, central domain, and carboxy-terminal domain. The amino-terminal recognizes and binds to substrates, the central ATPase domain contains an ATP-binding motif, whereas the carboxy-terminus contains the catalytic serine-lysine dyad for proteolysis (10–12).

Worldwide, research is being conducted on the use of bioremediation to effectively decontaminate nitroaromatics, and enzymes that facilitate the reduction process are of interest to biotechnologists. The possible role of Lon protease in the metabolism of 2,4-DNP is an unexplored area of research. In this study, we aim to decipher the roles of *E. coli*-encoded Lon protease in the metabolism of 2,4-DNP. The study highlights the pivotal role of Lon protease in conversion of xenobiotic nitroaromatic 2,4-DNP by *E. coli*. The enhanced conversion observed in the Δ*lon* strain highlights the influence of Lon on nitroreductase activity, likely mediated through the MarA regulatory system. This work provides valuable insights into the biological pathways involved in nitroaromatic compound degradation by characterizing the distinct conversion products and elucidating the regulatory mechanisms.

## RESULTS

### UV-visible spectral pattern of conversion products of 2,4-DNP differs in wild-type and Δ*lon* strains

**Fig 1:**
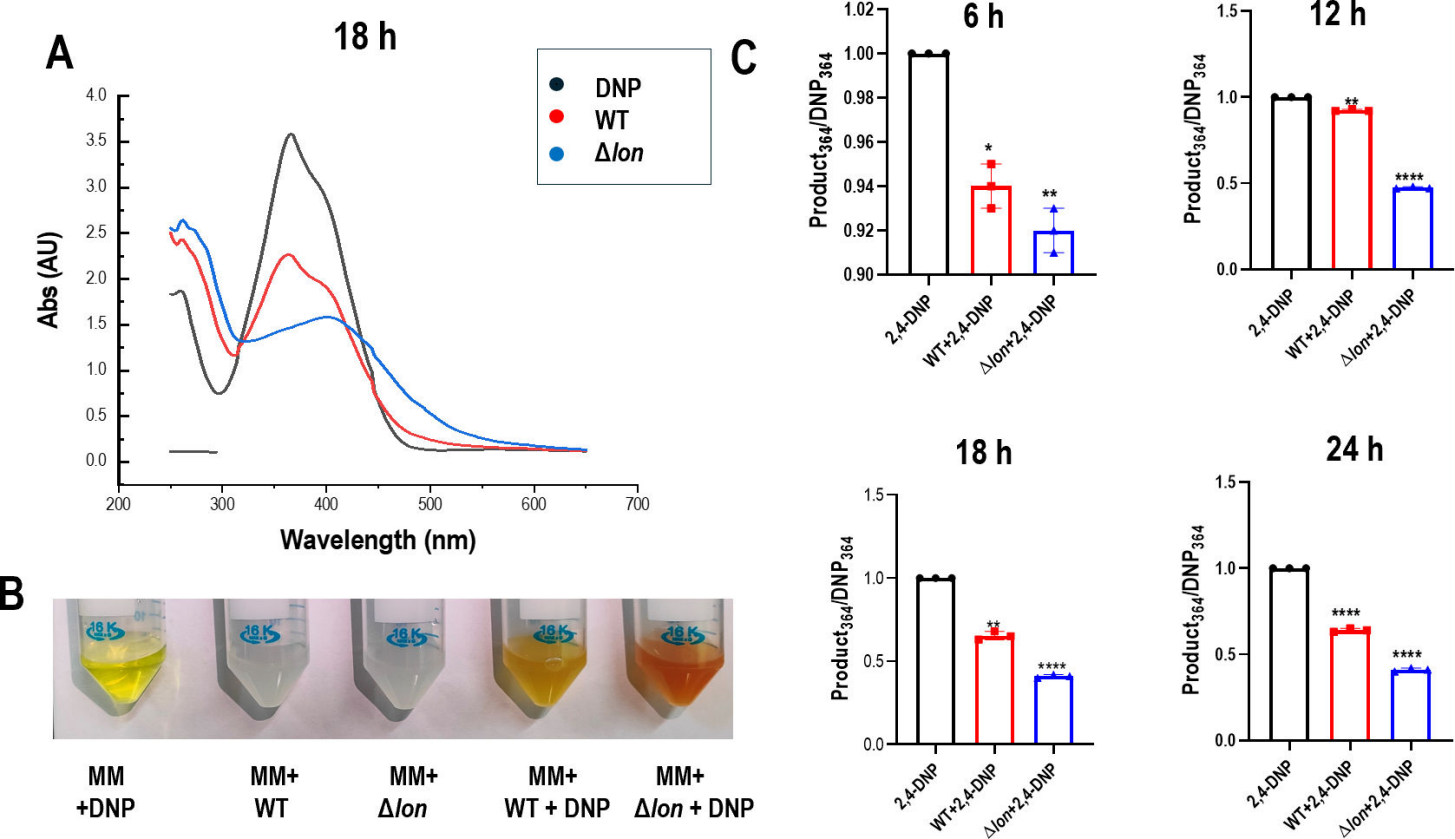
A reddish-brown-colored compound is produced in higher amounts in the Lon protease-deficient (Δ*lon*) strain grown in minimal media in the presence of 2,4-DNP. *E. coli* MG1655 wild-type and Δ*lon* strains were cultured for 18 h at 37°C and 160 rpm in the presence of 0.5 mM of 2,4-DNP. Culture supernatants were collected from bacterial culture grown in the absence and presence of 0.5 mM 2,4-DNP. (**A**) UV-visible spectrum of the supernatant after diluting 1:2. (**B**) Representative image showing conversion product formed post 18 h of treatment. (**C**) Quantification of the spectrum was performed for 6, 12, 18, and 24 h. The data are representative of three independent experiments plotted as mean ± SD. Statistical analysis was performed using two-way ANOVA and one-way ANOVA for (**C**), where * indicates *P* < 0.05, **P ≤ 0.01, and ****P ≤ 0.0001.

During our studies with uncouplers 2,4-DNP and CCCP, where we explored the role of Lon protease in imparting resistance (13), we observed the formation of a reddish-brown color in wild-type (WT) and Δ*lon* strains with 2,4-DNP, and the intensity of color in the Δ*lon* strain was higher. WT and Δ*lon* strains incubated with 2,4-DNP (yellow color) showed change in the color of the supernatant from 12 h post-treatment, where both the supernatants showed dark orange color. However, from the 18th h post-treatment, the color of the supernatant of the Δ*lon* strain changed to an intense reddish-brown compared to the supernatant of the WT strain. These observations were investigated in greater detail, and bacterial cultures were grown in Minimal Media (MM) with or without 0.5 mM 2,4-DNP for 6, 12, 18, and 24 h, and cells were pelleted down at 10,000 × *g* for 10 mins. WT and Δ*lon* strains were also grown in the presence of two different concentrations of 2,4-DNP: 0.25 mM (low) and 0.75 mM (high). The supernatant was collected and filtered, and UV-vis spectra of the supernatant were recorded in a UV-vis spectrophotometer. The reduction in the concentration of 2,4-DNP during the conversion was quantified using the formula mentioned in Materials and Methods, and the ratios were plotted in a graph (Fig. 1; Fig. S1). The spectral pattern of 2,4-DNP was similar to the previously reported pattern (14, 15), with a peak maximum of 364 nm. The spectral peak of Δ*lon* showed a signature peak shift when treated with 0.25 mM but failed to show the peak shift with 0.75 mM, mostly due to toxicity at higher doses; thus, 0.5 mM was selected as the optimum dose for further experiments involving spectral profiling (Fig. S1D and E). There was a change in the peak maxima for the new compounds formed when 0.5 mM 2,4-DNP was inoculated with the Δ*lon* strains from 12 h post-treatment (Fig. S1B and C); however, a significant difference in the peak pattern of the supernatant obtained from the two strains under investigation compared to 2,4-DNP spectra was observed at 18 h post-treatment (Fig. 1A). A reduction in the absorption of 2,4-DNP peak maximum could be observed in the WT supernatant, and a red shift could be observed in the supernatant from the Δ*lon* strain. The WT supernatant showed a peak of 364 nm, whereas the mutant supernatant gave a peak of 394 nm.

### The higher conversion product seen in the Δ*lon* strain is not due to the higher growth of the mutant in the presence of 2,4-DNP

Overall, it appeared that the Δ*lon* strain could convert 2,4-DNP (yellow) to a reddish-brown color in the growth media faster than the WT strain. The effect could be due to the higher growth of Δ*lon* strain in the presence of 2,4-DNP. To address this aspect, both bacterial strains were grown in 50 mL MM with and without 0.5 mM 2,4-DNP, and the optical density at 600 nm and colony-forming unit/mL (CFU/mL) was measured at regular intervals from 0 to 24 h. A dose screening was done to find the optimal dose for the growth experiment with 2,4-DNP in MM, and 0.5 mM was found to be the optimal dose to study the growth kinetics (Fig. S2A). The growth rate of the Δ*lon* strain was lower than that of WT in the presence of 2,4-DNP in MM from 3 h post-treatment (Fig. S2B and C). Thus, the greater conversion product seen in Δ*lon* was not due to higher growth of this knock-out strain. It is likely that the difference in conversion observed was due to changes in the metabolism of 2,4-DNP in the absence of Δ*lon*.

### *trans* complementation of *lon* increases the growth of Δ*lon* but reduces the amount of reddish-brown color in the presence of 2,4-DNP

**Fig 2:**
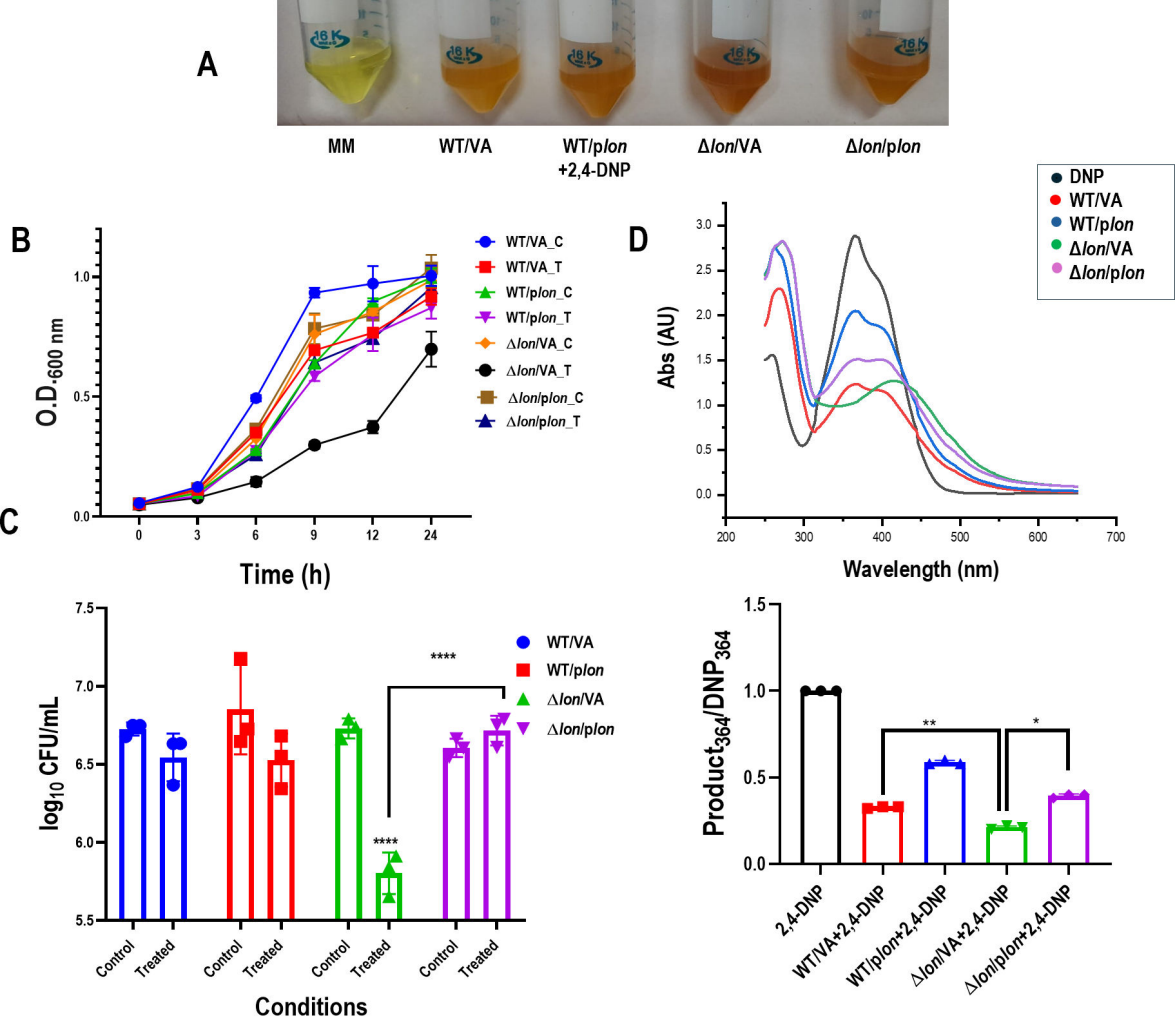
*trans c*omplementation of *lon* increases the growth of Δ*lon* in the presence of 2,4-DNP but lowers the amount of the reddish-brown colored compound. *E. coli* WT /VA, WT /p*lon,* Δ*lon/*VA, and Δ*lon/*p*lon* were cultured in the presence of 0.5 mM 2,4-DNP for a period of 18 h at 37°C and 160 rpm. (**A**) Representative image showing conversion product formed post 18 h of treatment. (**B**) Growth of the strains during exposure to 0.5 mM 2,4-DNP is shown. (**C**) CFU count is shown 6 h post-exposure. (**D**) UV-visible spectrum of the supernatant after diluting 1:2 with a graph showing the quantification of the spectrum. C represents the control group, and T represents the treated group. The data are representative of three independent experiments plotted as mean ± SD. Statistical analysis was performed using two-way ANOVA and one-way ANOVA for (**D**), where * indicates *P* < 0.05, **P ≤ 0.01, and ****P ≤ 0.0001.

*trans* complementation was performed to confirm the *lon* dependency of the phenotypes observed with the strain. Growth and spectral patterns of the growth supernatant of strains transformed with the empty vector and plasmid with the gene of interest were investigated. *trans* complementation of *lon* partially rescued the peak shift observed in Δ*lon*/VA (plasmid vector transformed strain) to the WT spectral pattern discussed in the previous result (Fig. 2). Quantifying the spectra further confirms the partial rescue of 2,4-DNP drop-in Δ*lon*/p*lon* (plasmid containing the *lon-*transformed strain) . The growth reduction induced by 2,4-DNP in Δ*lon*/VA was also rescued in Δ*lon*/p*lon* (Fig. 2B and C). WT/p*lon,* the over-expression system of *lon,* showed a lesser decrease in 2,4-DNP (Fig. 2D). All these confirm that the growth phenotype and the spectral pattern observed were *lon*-dependent.

### The conversion of 2,4-DNP into the reddish-brown color is MarA-dependent

**Fig 3:**
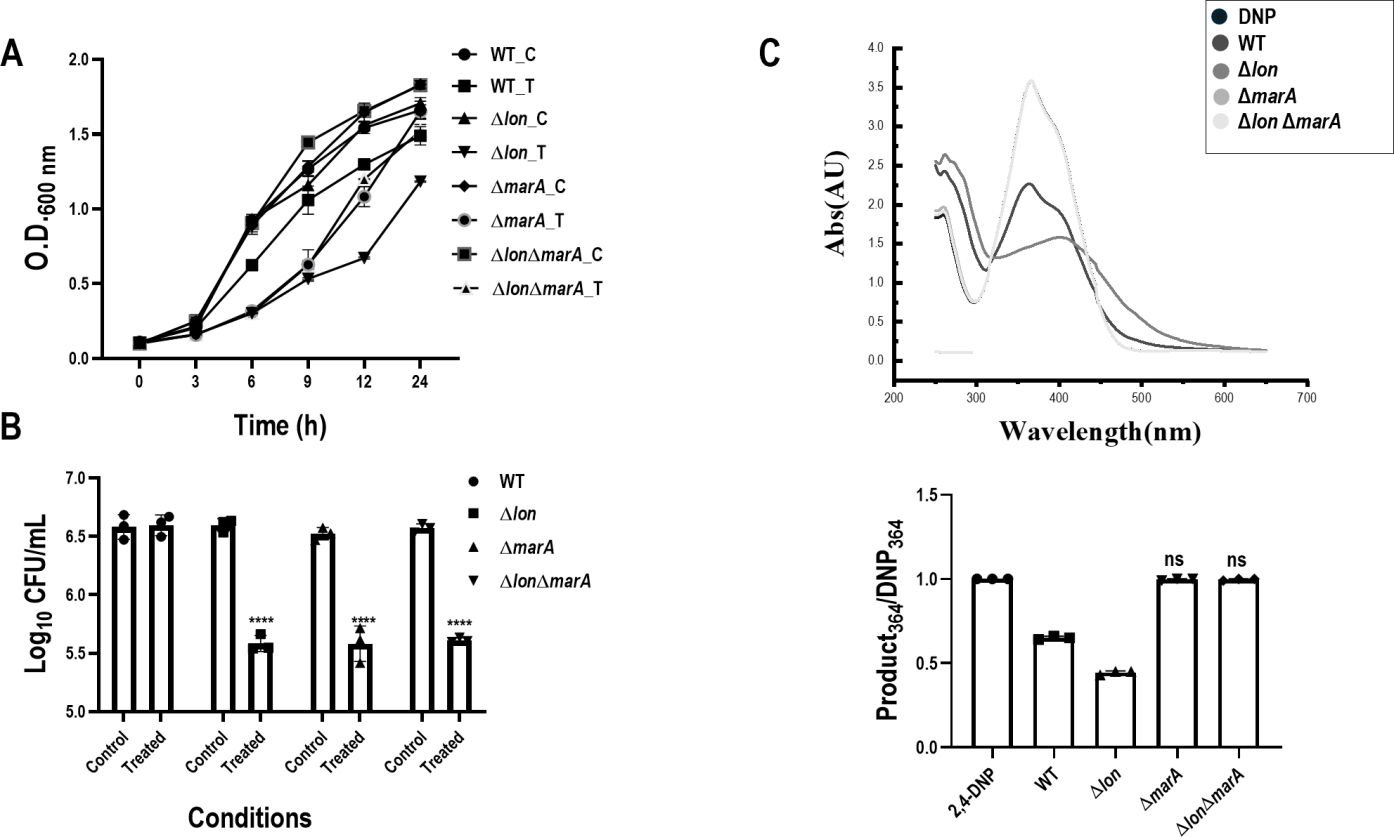
The *marA* mutant strain fails to convert 2,4-DNP to the reddish-brown-colored compound. *E. coli* WT, Δ*lon*, Δ*marA*, and Δ*lon* Δ*marA* were cultured in the presence of 0.5 mM 2,4-DNP for a period of 18 h at 37°C and 160 rpm. (**A**) The growth of the strains during exposure to 0.5 mM 2,4-DNP is shown. (**B**) CFU count is 6 h post-exposure. (**C**) UV-visible spectrum of the supernatant after diluting 1:2 with a graph showing the quantification of the spectrum. C represents the control group, and T represents the treated group. The data represent three independent experiments, plotted as the mean ± SD. Statistical analysis was performed using two-way ANOVA for (**B**) and one-way ANOVA for the graph showing quantification of the spectrum (**C**), where * indicates *P* < 0.05 and ns indicates not significant.

**Fig 4:**
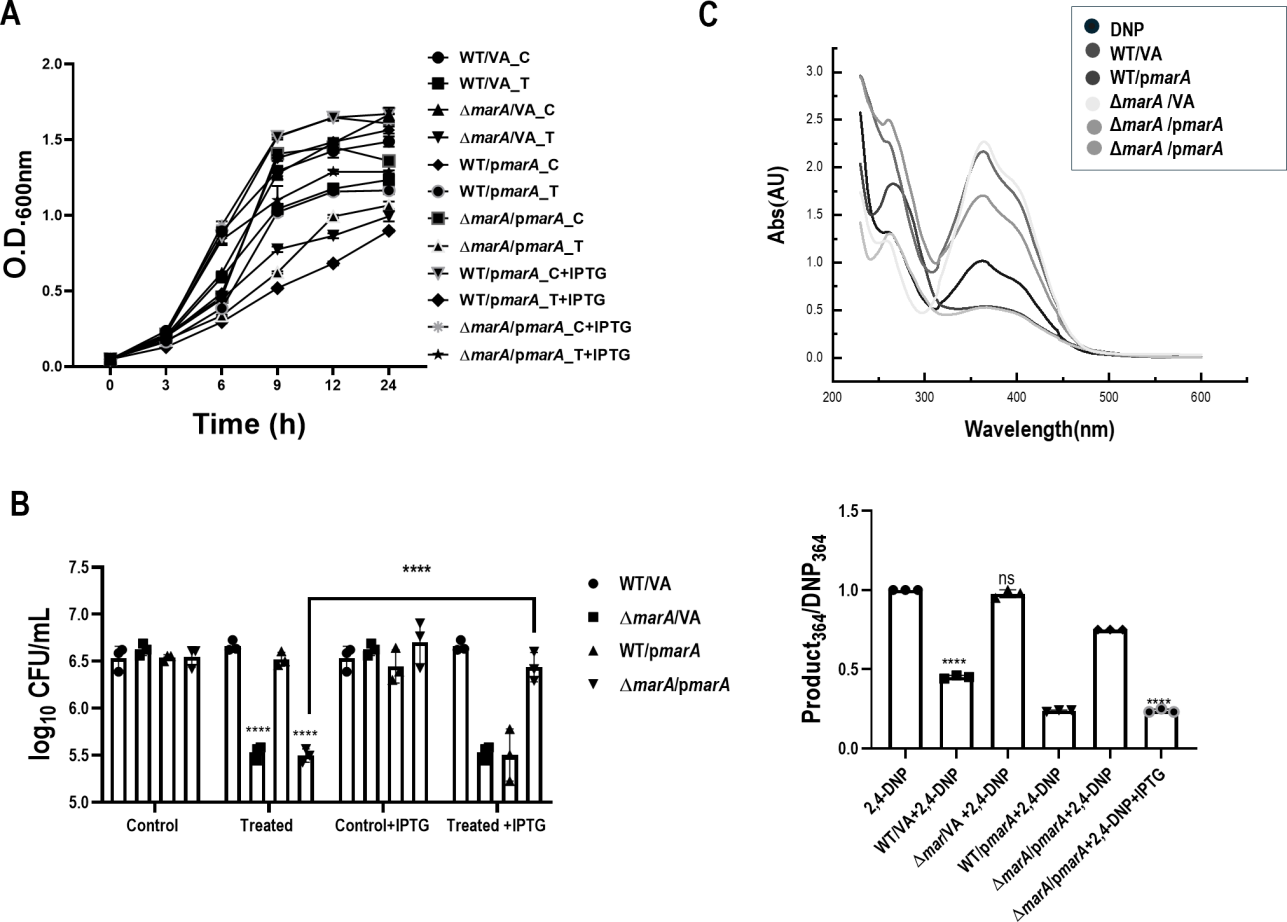
*trans* complementation confirms the *marA* dependency of 2,4-DNP metabolism. *E. coli* WT/VA, WT/ /p*marA*, Δ*marA*/VA, and Δ*marA*/p*marA* were cultured in the presence of 0.5 mM 2,4-DNP for a period of 18 h at 37°C and 160 rpm. (**A**) The growth of the strains during exposure to 0.5 mM 2,4-DNP is shown. (**B**) CFU count is shown 6 h post-exposure. (**C**) The UV-visible spectrum of the supernatant after diluting 1:2 with a graph showing quantification of the spectrum. C represents the control group, and T represents the treated group. The data represent three independent experiments, plotted as the mean ± SD. Statistical analysis was performed using two-way ANOVA for (**B**) and one-way ANOVA for the graph showing quantification of the spectrum (**C**), where * indicates *P* < 0.05 and ns indicates not significant.

The Lon protease has various substrates, and several strains lacking many of these substrates are already available in our laboratory (13). Single knockouts of *marA, rob,* and *soxS* were screened for their ability to convert 2,4-DNP to the reddish-brown color (Fig. S3). The Δ*rob* and Δ*soxS* strains converted 2,4-DNP to the reddish-brown color, although some changes were seen in the Δ*rob* strain compared to WT (Fig. S3C). However, the Δ*marA* strain completely failed to convert 2,4-DNP to the reddish color. Also, the Δ*lon*Δ*marA* strain failed to convert 2,4-DNP to the reddish-brown color, which showed the major dependence of the conversion phenomenon on *marA* (Fig. 3). To further confirm the phenomenon, *trans* complementation of *marA* was generated, and the WT/VA and Δ*marA*/VA strains showed the phenotypes of WT and Δ*marA*, respectively, in terms of conversion and growth. WT/p*marA* showed higher conversion, depicting that higher amounts of *marA* led to greater conversion of the substrate 2,4-DNP. Δ*marA*/p*marA* rescued the conversion and growth reduction phenotype observed in Δ*marA* compared to the WT phenotype (Fig. 4). All these results confirm that the conversion of 2,4-DNP to the reddish-brown color is *marA-*dependent.

### The nitroreductase deletion mutants are less efficient in converting 2,4-DNP to the reddish-brown color

**Fig 5:**
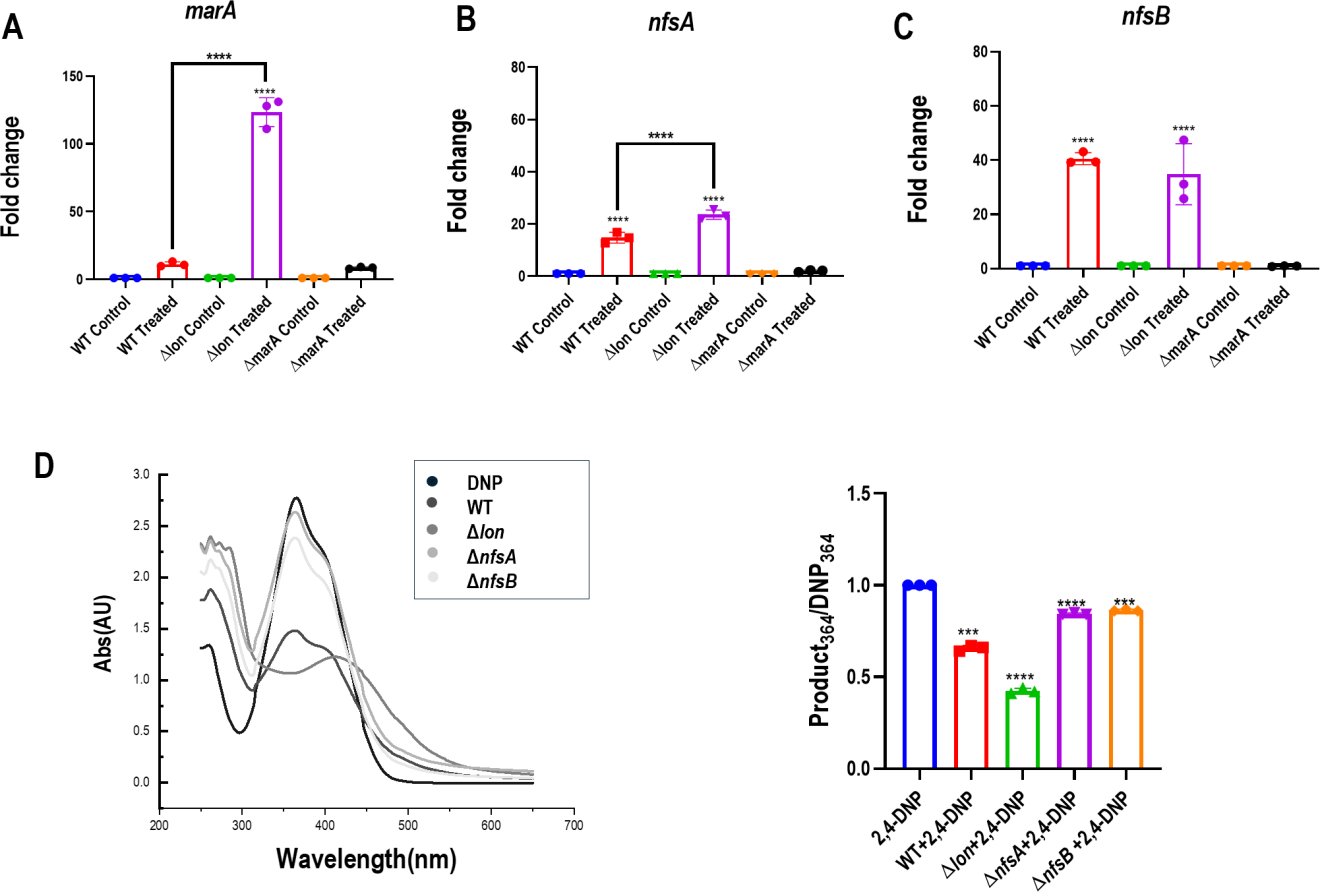
2,4-DNP induces the expression of *marA*, *nfsA,* and *nfsB* in both WT and ∆*lon* strains but not in the Δ*marA* strain. The strains were grown for 3 h at 37^○^C, and then 2,4-DNP was added for the treated condition; cells were harvested 3 h post-treatment and processed for RNA isolation. Fold changes in transcript levels of (**A**) *marA*; (**B**) *nfsA*; (**C**) *nfsB* in WT, ∆*lon,* and Δ*marA* strains were determined by quantitative real-time PCR. *gapA* was used as the reference gene. *E. coli* WT, Δ*lon*, Δ*nfsA,* and Δ*nfsB* were cultured in the presence of 0.5 mM 2,4-DNP for a period of 18 h at 37°C and 160 rpm. (**D**) UV-visible spectrum of the supernatant after diluting 1:2 with MM, and graph showing quantification of the spectrum. The data are representative of three independent experiments with mean ± SD, where * indicates *P* < 0.05, ***P ≤ 0.001, and ****P ≤ 0.0001. Statistical analysis was performed for each strain relative to its untreated control. Comparison between the strains is indicated wherever significant.

Bacterial nitroreductases (NfsA and NfsB) play a major role in the metabolism of nitroaromatic (16, 17). Both *nfsA* and *nfsB* (nitroreductase genes) have a MarA box (Fig. S4), which induces the expression of the two genes when the *mar* operon is derepressed (18). Since the roles of *lon* and *marA* were confirmed, we evaluated the expression of genes encoding these nitroreductases and their association with *marA* in the context of 2,4-DNP metabolism. The levels of *marA*, *nfsA,* and *nfsB* were upregulated with 2,4-DNP treatment in both WT and Δ*lon* strains; however, the upregulation was not observed in Δ*marA* post-treatment with 2,4-DNP (Fig. 5). These results establish the direct link of *marA* with the induction of nitroreductase genes upon exposure to 2,4-DNP. To further understand the possible functional roles of the nitroreductase genes in the metabolism of 2,4-DNP, *nfsA* and *nfsB* knock-outs were screened. Δ*nfsA* and Δ*nfsB* showed a slight reduction in the amounts of 2,4-DNP in the supernatant compared to 2,4-DNP in the MM control (Fig. 5D and E). It is possible that in the absence of one nitroreductase, the other one is compensating for the function. These results confirmed the roles of *marA* and both nitroreductases for 2,4-DNP conversion.

### 4-Amino-2-nitrophenol is the compound corresponding to the peak m/z = 153.03 in the LC-MS spectrum

**Fig 6:**
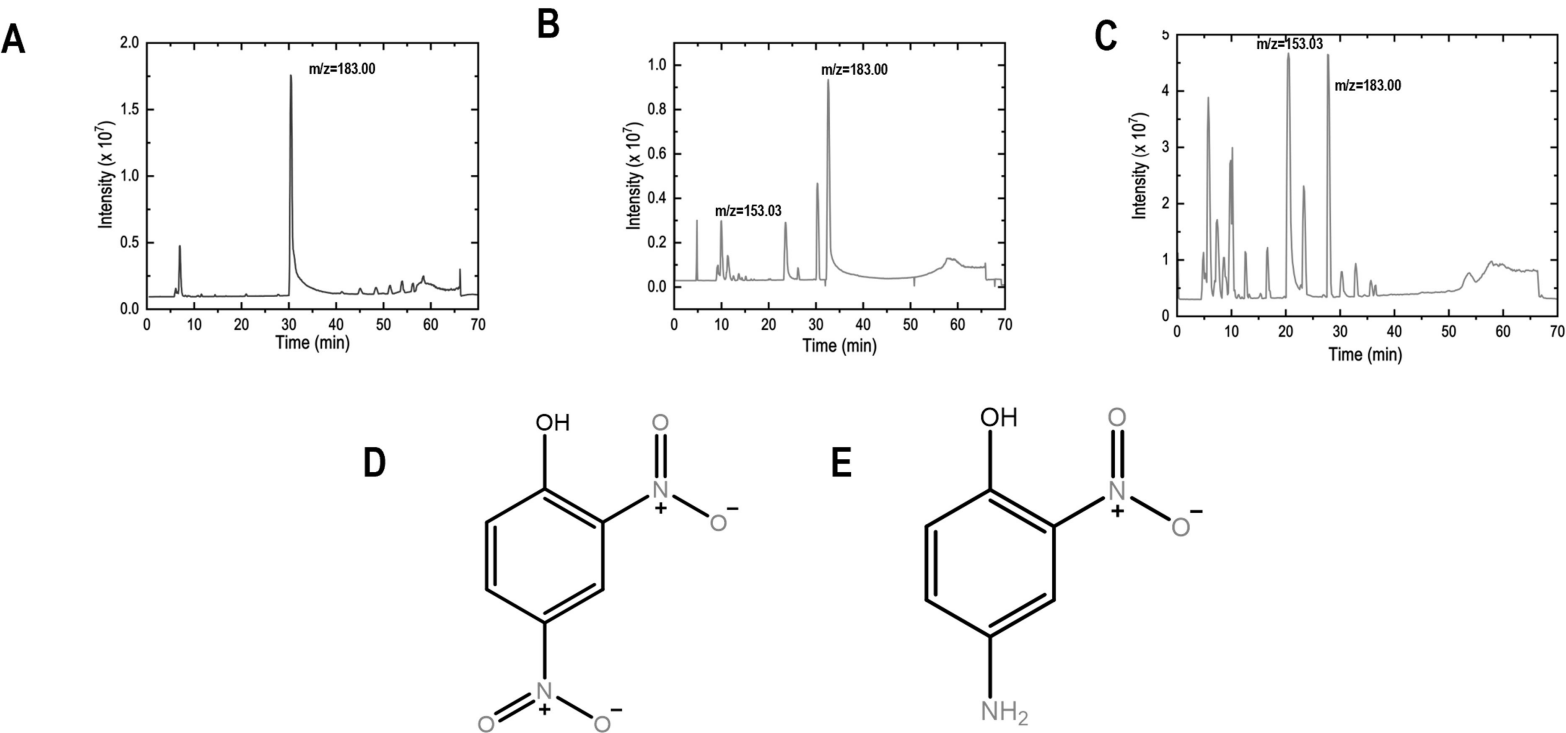
A compound corresponding to the peak *m/z* = 153.03 is confirmed to be 4-amino-2-nitrophenol. The strains were grown for 18 hours at 37^○^C in the presence of 2,4-DNP; the supernatant was collected and processed for LC-MS. (**A**) The suspension of 2,4-DNP clearly showed a single peak designating an *m/z* value of 183.0045, which, upon (**D**) analysis and fragment search, was confirmed to be from 2,4-DNP. (**B**) WT strain showed two major peaks at *m/z* = 183.0045 and 153.0310, and (**E**) peak of *m/z* = 153.0310 was confirmed to be 4-amino-2-nitrophenol. (**C**) Δ*lon* strain showed many major peaks, out of which two peaks corresponding to *m/z* = 183.0045 and 153.031 could be identified as 2,4-DNP and 4,2-ANP, respectively.

To identify the conversion product, we analyzed the supernatant using LC-MS and chose this strategy due to its high accuracy. The chromatogram obtained from LC-MS samples was overlapped, and the common and unique peaks were identified and marked. The intensity of the peaks was also analyzed to understand the conversion product of 2,4-DNP and other metabolites being released into the growth suspension. The suspension of 2,4-DNP clearly showed a single peak corresponding to an *m/z* value of 183.0045, which, upon analysis and fragment search, was confirmed to be from 2,4-DNP (Fig. 6A and E). This served as a control and validated the experimental flow, and the peak intensity was 2 × 10^7^. The chromatogram obtained from the growth suspension of the WT strain showed three major peaks at *m/z* = 183.0045, 153.0310, and 329. 0550 (Fig. 6A). The first peak was identified as corresponding to 2,4-DNP but with a lower intensity of 1.2 × 10^7^ as 2,4-DNP was being metabolized during bacterial growth. The second peak of *m/z* = 153.0310 had two hits with a final score of 1.0, which were 4-amino-2-nitrophenol and 2-amino 4-nitrophenol, and the peak intensity was 0.3 × 10^7^. The Δ*lon* strain showed a few major peaks, out of which two peaks corresponding to *m/z* = 183.0045 and 153.031 could be identified as 2,4-DNP and 4,2 -ANP, respectively (Fig. 6C). The peak intensities of 2,4-DNP and 4,2 -ANP were around 0.1 × 10^7^ and 0.7 × 10^7^ respectively. Since LC-MS relies solely on a molecule’s mass/charge ratio, isomeric forms cannot be differentiated. Since the nitro group in the meta position of 2,4-DNP cannot undergo reduction due to the electron-withdrawing nature of the hydroxyl group in the ortho position, the compound was confirmed to be 4-amino-2-nitrophenol (Fig. 6D). The third peak of *m/z* = 329.0550 did not give any hits from the KEGG database. Since the identified compound is reddish-brown, the conversion product can be confirmed as 4-amino-2-nitrophenol.

### 4,2-ANP induces phenotypic antibiotic resistance in an *acrB-*dependent manner

We found that 2,4-DNP is reduced to 4,2-ANP by *E. coli*. In a continuous culture with a regular flow of nutrients, the conversion product can affect the metabolism and be a possible inducer of phenotypic antibiotic resistance. To answer the above question, we screened WT with different doses of 4,2-ANP and compared the growth with 2,4-DNP in LB. As previously reported, 2,4-DNP reduced the growth of the WT strains (13); however, no significant reduction in growth was observed by 4,2-ANP (Fig. S5A). Broth dilution MIC was used to test the ability of 4,2-ANP to induce antibiotic resistance against different classes of antibiotics and compared it with that of 2,4-DNP: ciprofloxacin, a bactericidal antibiotic inhibiting DNA replication; tetracycline, a bacteriostatic antibiotic inhibiting protein synthesis; and ampicillin, a bactericidal antibiotic targeting cell wall synthesis. Both 2,4-DNP and 4,2-ANP induced antibiotic resistance against ciprofloxacin, tetracycline, and ampicillin. 2,4-DNP was more potent in inducing resistance against ciprofloxacin, whereas 4,2-ANP was more potent in inducing tetracycline resistance, and both compounds were equally potent in inducing resistance against ampicillin (Fig. S5B, C, and D).

**Fig 7:**
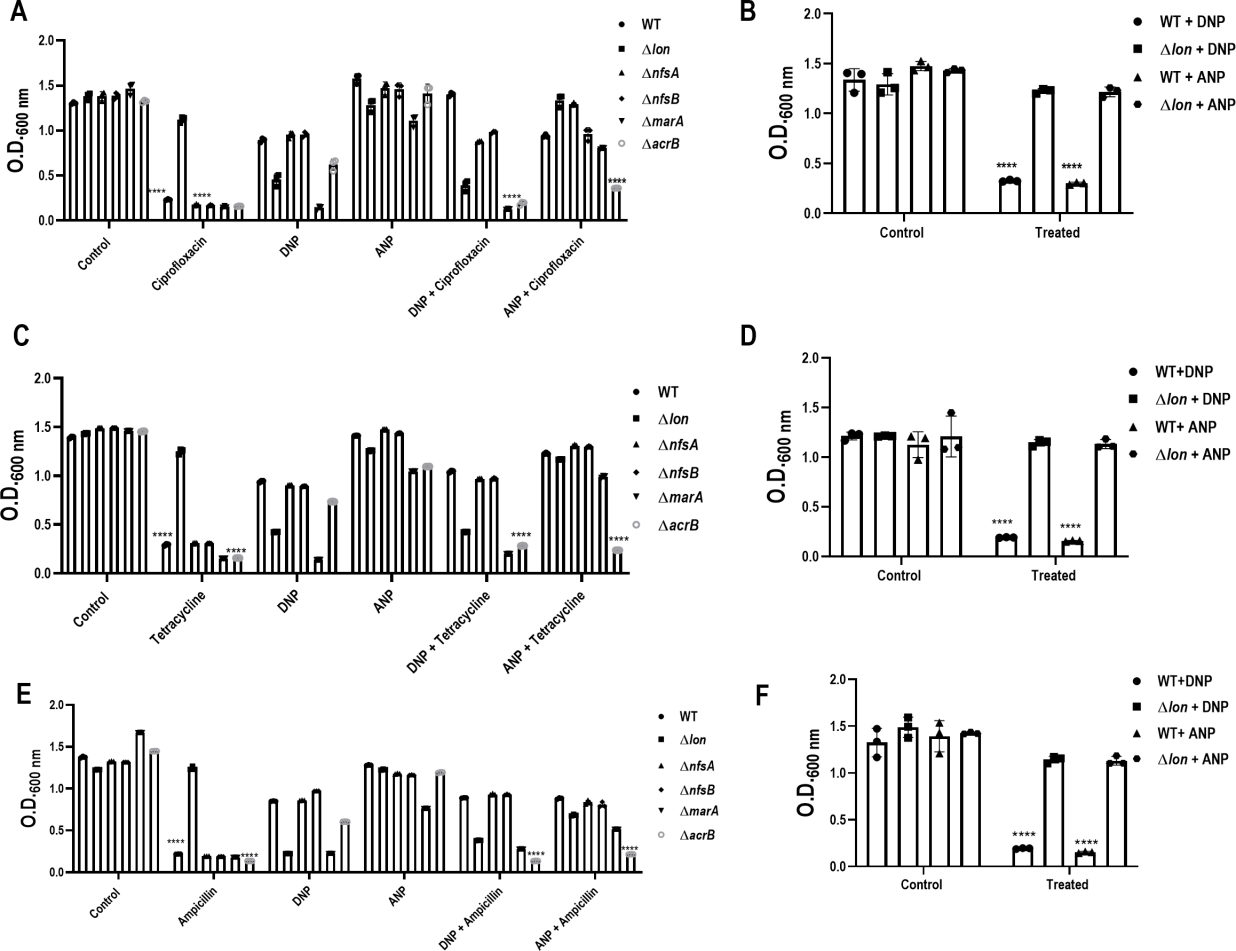
4,2-ANP induces phenotypic antibiotic resistance in an *acrB*-dependent manner. *E. coli* WT, Δ*lon*, Δ*marA*, Δ*acrB*, Δ*nfsA*, and Δ*nfsB* strains were cultured in the presence or absence of 2,4-DNP and 4,2-ANP with (**A**) ciprofloxacin; (**B**) tetracycline; (**C**) ampicillin for 6 h at 37 ˚C and 160 rpm in LB. *E. coli* WT and Δ*lon* strains were grown in the presence of 0.5 mM 2,4-DNP or 0.5 mM 4,2-ANP and indicated antibiotics, washed with PBS normalized to OD2, and cultured with the corresponding antibiotics (**D**) ciprofloxacin; (**E**) tetracycline; (**F**) ampicillin, and the data are represented as a bar plot. The data are representative of at least three independent experiments plotted as mean ± SD. * indicates *P* < 0.05, ****P ≤ 0.0001. Statistical analysis was performed for each strain relative to its untreated control. Comparison between the strains is indicated wherever significant.

 To elucidate the pathway by which 4,2-ANP induces antibiotic resistance, we performed a growth assay with different knockouts. 2,4-DNP-induced resistance against ciprofloxacin and tetracycline was dependent on *marA* and *acrB,* as previously shown (13). Resistance induced by 2,4-DNP against ampicillin was explored in this study and found to be also dependent on both *marA* and *acrB*. 4,2-ANP also induced resistance against the antibiotics in an *acrB-*dependent manner since the Δ*acrB* failed to rescue the growth reduction induced by antibiotics in the presence of 4,2-ANP. On the other hand, the induction of resistance by 4,2-ANP failed to show dependence on *marA*. Strains lacking *lon* showed resistance against all the antibiotics. Since the role of nitroreductases was established in the conversion of 2,4-DNP to 4,2-ANP, we studied their role in induction of antibiotic resistance. The nitroreductases failed to show any role in the induction of resistance (Fig. 7A, B and C). Finally, to confirm the phenomenon observed is phenotypic antibiotic resistance, a transient form of resistance where bacteria exhibit resistance against antibiotic only in the presence of a cue/inducer, we grew WT and Δ*lon* strains in the presence of antibiotics and inducer for 6 h, washed the cells with PBS, normalized the culture to OD2, and inoculated 0.2% of OD2 culture in the presence of antibiotic alone and allowed it to grow for 6 h. All three antibiotics induced growth reduction in the WT strain, whereas the Δ*lon* strain was resistant against these antibiotics, confirming the phenomenon observed is indeed phenotypic antibiotic resistance (Fig. 7D, E and F).

## DISCUSSION

This work details the roles of Lon protease and the MarA transcription in the conversion of 2,4-DNP to 4,2-ANP and the identification of the latter as an inducer of phenotypic antibiotic resistance. Previously published reports from our group have shown that the *lon* deletion strain is sensitive to 2,4-DNP stress due to higher amounts of *marA* in nutrient-rich media (13). A similar phenotype was noticed when ∆*lon* was subjected to different doses of 2,4-DNP in minimal media. A more intense reddish-brown color was observed in the tubes from the 18th hour, even with lesser growth than WT (Fig. 1). UV-vis spectroscopy was used to analyze the change in the spectral pattern and identify the conversion product (19). A spectrum with a peak maximum at 364 nm was observed for 2,4-DNP, similar to previous reports (15). The WT supernatant showed similar peak maxima with lesser absorbance, which indicated the metabolism of the substrate, i.e., 2,4-DNP. However, the Δ*lon* supernatant showed a new spectral pattern with a bathochromic shift and a peak maximum at around 410 nm (Fig. 1). Although a change in the peak pattern was observed, the final product could not be concluded from the spectral patterns and peak maxima due to the unavailability of a database. It is also possible that the peak maxima and pattern could not match the available data due to a mixture of compounds in the media hindering the recording of a clean compound spectrum. The growth reduction phenotype and the conversion pattern in Δ*lon* were also *trans* complementable (Fig. 2).

The bathochromic shift indicates a reduction reaction, which pointed out the involvement of nitroreductases and intrigued us to study the possible interaction of Lon and nitroreductases in the context of 2,4-DNP metabolism. Nitroreductases in bacteria are flavoenzymes with NADH as an electron donor that catalyze the reduction of the nitro group in flavins, quinones, nitro heterocyclic, and nitroaromatic compounds (20). These enzymes have been widely studied due to their potential application in bioremediation and biomedicine (21). Although most nitroreductases have been purified and their biochemical properties characterized, their physiological roles remain obscure. Two types of bacterial nitroreductases have been described based on their response to oxygen: i) oxygen-insensitive or type I nitro and ii) oxygen-sensitive or type II nitroreductases (16, 17). Their phylogenetic analysis suggests that the type I nitroreductase can be classified into two families, NfsA and NfsB, dependent on NADPH and NADPH or NADH, respectively, as the electron donor. In *E. coli,* the *nfsA* and *nfsB* genes code for 27 and 24 kDa proteins termed major and minor nitroreductases, respectively, which are dissimilar in their amino acid sequences (16, 22, 23). Bacterial nitroreductases are believed to be regulated by *marRAB* and *soxRS* operon systems (18, 24). Numerous studies have shown that MarA, Rob, and SoxS are closely related and can bind to a degenerate sequence known as the *mar* box, overlapping the genes they regulate (25). The nitroreductase genes also have a *mar* box in their upstream sequence (24). Similar to sodium salicylate, 2,4-DNP can also bind to MarR and initiate the transcription of *marA* (26). It is also known that Lon protease can maintain the homeostasis of these three transcription factors, and the absence of Lon can lead to higher amounts of transcription factors (27). Therefore, we screened these genes (*marA*, *rob,* and *soxS*) for their ability to convert 2,4-DNP and found that the conversion was entirely *marA-*dependent since Δ*marA* cells failed to show any reduction in the intensity of 2,4-DNP (Fig. 3). Also, the Δ*lon*Δ*marA* strain showed no conversion, and the spectral pattern overlapped with that of 2,4-DNP alone (Fig. 3), like Δ*marA*, confirming that MarA was responsible for the phenotype observed in the strain lacking *lon*; i.e., higher levels of MarA in Δ*lon* lead to the higher conversion of 2,4-DNP. Additionally, qPCR showed the upregulation of the expression of nitroreductase genes (*nfsA* and *nfsB*) in both WT and Δ*lon* upon treatment with 2,4-DNP*,* and the upregulation was higher in Δ*lon*. However, no upregulation of the expression of nitroreductase genes was observed in Δ*marA* cells upon treatment with 2,4-DNP (Fig. 5A–C). These results confirm the interplay between Lon, MarA, *nfsA,* and *nfsB* upon exposure to 2,4-DNP. Since both nitroreductases showed upregulation, we could not conclude the key nitroreductase involved in the metabolism of 2,4-DNP. To resolve this, we utilized the knock-out strains of *nfsA* and *nfsB* and screened them for their ability to convert 2,4-DNP. To our surprise, both knockouts failed to show conversion of 2,4-DNP (Fig. 5D).

**Fig 8:**
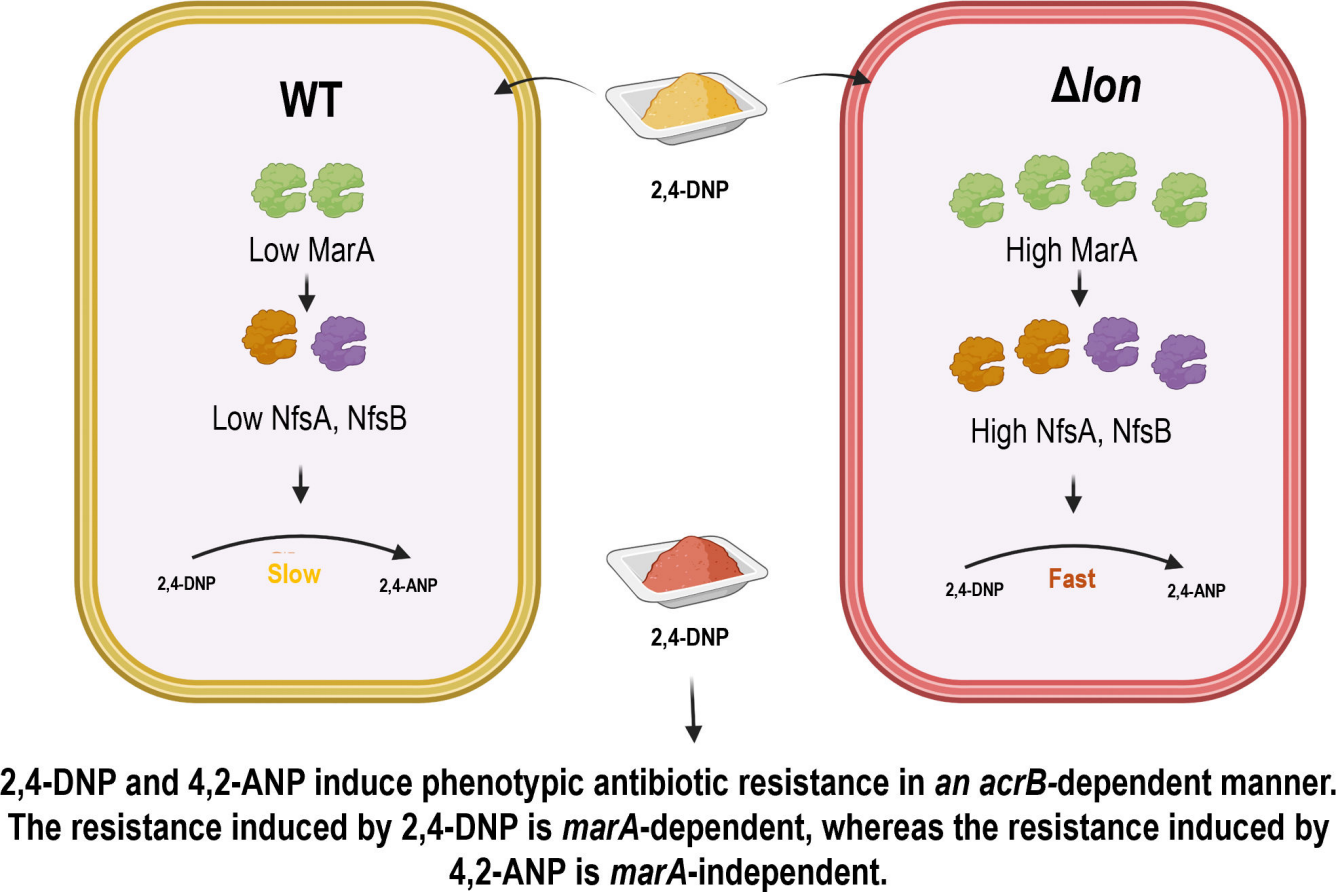
A model representing the roles of Lon protease and its substrate MarA in the generation of 4,2-ANP, which induces phenotypic antibiotic resistance. The roles of Lon protease in degrading the excess amount of MarA, which induces the activity of nitroreductases NfsA and NfsB in the metabolism of 2,4-DNP, are shown. The study identifies 4,2-ANP as a novel inducer of phenotypic antibiotic resistance. Also, the key differences between DNP and ANP in inducing phenotypic antibiotic resistance are shown.

A high-throughput assay, i.e. LC-MS, was used to determine the compound formed in the growth suspension (28). LC-MS was carried out from the growth suspension, and the presence of 4,2-ANP was confirmed in both the WT and Δ*lon* strains. There were many other compound peaks in the LC-MS spectrum, but most remained unresolved, mainly carbohydrate or fatty acid molecules that are mostly not relevant to this study. The concentration of 4,2-ANP was found to be higher in the Δ*lon* strain, together with a reduction in the concentrations of 2,4-DNP (Fig. 6). 4,2-ANP is a commonly used noncarcinogenic compound in hair color dyes and is considered safe for humans (29). 4,2-ANP has been previously studied in various aspects, including safety for usage in hair color dyes, induction of mutation in *Salmonella* Typhimurium, percutaneous absorption in human and monkey skin, and effects on the metabolism of rats (29–32). However, 2,4-DNP metabolism by *E. coli* and induction of phenotypic antibiotic resistance by 4,2-ANP remains an unexplored area. Compared with 2,4-DNP, 4,2-ANP was less potent in reducing the growth and induction of resistance against ciprofloxacin, but it was more potent than 2,4-DNP in inducing resistance against tetracycline (Fig. 7). In our previous study, the roles of *marA* and *acrB* in inducing resistance against antibiotics in the presence of 2,4-DNP were established (13). In this study, we demonstrate that 4,2-ANP is less potent and induces phenotypic antibiotic resistance in an *acrB*-dependent manner (Fig. 7 and Fig. 8).

*E. coli* is one of the common microorganisms in fecally contaminated sewage and often interacts with several pollutants (33, 34), but *E. coli’*s versatility in bioremediation remains less explored, leading to a large gap in the field. *E. coli*’s potential can be fully used if we expand our knowledge about how an organism metabolizes different things (35). Nitroaromatic compounds like 4,2-ANP are widely used in industrial applications, including dye production. The findings suggest that their environmental presence may inadvertently influence microbial communities, impacting ecosystems and potentially human health. Long-term exposure to nitroaromatics can lead to mutations (36), and mutations in *lon*, *marA*, and *acrB* may lead to the development of antibiotic-resistant strains in the environment (37). Previous studies have shown that bacteria exposed to a similar compound, dinitrotoluene (DNT), induce mutagenic oxidative stress, leading to an evolutionary benefit in terms of biodegrading DNT through an SOS-independent pathway (38, 39). The evolutionary trajectory of a bacterium toward the development of xenobiotic degradation pathways is often influenced by its background metabolism (40); also, stress is known to induce ROS. Frequent exposure to DNP could lead to an evolutionary trajectory that favors DNP degradation. This study calls for immediate attention to studying the effects of long-term exposure to a nitroaromatic antibiotic commonly used against uropathogenic *E. coli*.

This study adds to the limited body of knowledge regarding the metabolic and phenotypic effects of nitroaromatic compounds on bacteria. It introduces a new perspective on how such compounds interact with microbial systems, paving the way for further research into metabolic pathways and regulatory mechanisms. These findings have broader implications for bioremediation, suggesting that strains lacking Lon protease or engineered to enhance nitroreductase activity could be optimized for environmental applications. These results contribute to our understanding of the microbial metabolism of toxic compounds and open avenues for sustainable solutions to industrial pollution through targeted biotechnological innovations. Also, this study emphasizes the need to carefully assess chemical pollutants and their unintended consequences on microbial resistance. Ultimately, this study lays the foundation for developing efficient microbial systems for detoxifying and bioremediation of harmful environmental pollutants.

## MATERIALS AND METHODS

### Bacterial strains and growth conditions

The bacterial strains used in this study are listed in Table 1. All the cultures were grown in Luria Bertani (LB) broth comprising tryptone (10 g/L; HiMedia, Mumbai, India), NaCl (10 g/L; HiMedia), and yeast extract (5 g/L; HiMedia) at 37 ^˚^C and 160 rpm. Overnight-grown cultures obtained from a single colony of the strains served as per-inoculum for all experiments. Antibiotics were used at 100  µg/mL ampicillin, 30  µg/mL chloramphenicol, and 50  µg/mL kanamycin (HiMedia). Cultures for experiments were carried out in MM containing 64 g/L Na_2_HPO_4_•7H_2_O, 15 g/L KH_2_PO_4_, 2.5 g/L NaCl, and 5.0 g/L NH_4_Cl. The optical density (O.D.) of all the strains was normalized to O.D. 2, which corresponds to 10^9^ CFU/mL, before all experiments, and 0.2% and 1% of these O.D. 2 cultures were used as the starting inoculum for all experiments in LB and MM, respectively, for all experiments. A UV-visible spectrophotometer (Tecan, Männedorf, Switzerland) determined growth by measuring the O.D. _600 nm_.

**TABLE 1:** List of strains used in the study

Sl.no	Strain	Genotype/feature	Reference
1	WT MG1655	λ, rph-1	Guyer et al. (41)
2	Δ*lon*	MG1655, *lon*::*cat*	Bhaskarla et al. (27)
3	Δ*marA*	MG1655, *marA*::*kan*	Baba et al. (42)
4	Δ*lon*Δ*marA*	MG1655, *lon*::*cat*, *marA*::*kan*	Bhaskarla et al. (27)
5	Δ*acrB*	MG1655, *acrB*::*kan*	Baba et al. (42)
6	WT/VA	Isogenic complement strain for WT with plasmid pQE60, Amp^r^	This study
7	WT/p*marA*	Isogenic complement strain for WT expressing *marA* under the T5 promoter present in plasmid pQE60, Amp^r^	This study
8	Δ*marA*/VA	Isogenic complement strain for Δ*marA* with plasmid pQE60, Amp^r^	This study
9	Δ*marA*/p*marA*	Isogenic complement strain for Δ*marA* expressing *marA* under the T5 promoter present in plasmid pQE60, Amp^r^	This study
10	*E. coli* Δ*lon*::*kan*	*lon*-deficient derivative of wild-type	Kind gift from Dr. Nishad Matange, IISER-Pune, India (43)
11	*E. coli* Δ*nfsA*	*nfsA*-deficient derivative of wild-type	Kind gift from Prof. Amit Singh, IISc Bengaluru, India
12	*E. coli* Δ*nfsB*	*nfsB*-deficient derivative of wild-type	Kind gift from Prof. Amit Singh, IISc Bengaluru, India

### Recording UV-visible spectrum

To record the conversion products’ UV-visible spectra, the strains were grown in 5 mL of MM media in the presence of 0.5 Mm 2,4-DNP for 24 h at 37 ˚C at 160 rpm. The cells were pelleted down at 10,000 × *g* for 10 mins, and the supernatant was collected in a fresh Falcon tube. The supernatant was filtered using a sterile syringe (BD, Belgium) with a polyvinylidene fluoride (PVDF) filter membrane of 0.22 µm pore size (GE Health Care UK Limited, Hertfordshire, UK). Subsequently, 1:2 and 1:4 dilutions of the filtered supernatant were prepared by diluting with MM media. A concentration of 0.5 mM of 2,4-DNP in MM filtered, post 24 h of incubation following all the conditions of the bacterial cultures, served as the reference. UV-visible spectra of the diluted supernatant solutions were recorded in a spectrophotometer (Spectramax M5e, Avantor, Pennsylvania, USA) using the software SoftMax pro-7. The spectra were plotted using the software Origin-2018.

### Quantification of the UV-visible spectrum

The reduction in the concentration of 2,4-DNP during the conversion was quantified by calculating the drop in the absorbance of the peak maxima of 2,4-DNP, i.e., 364 nm. The quantification was done using the formula: absorbance at 364 nm for the conversion product divided by absorbance at 364 nm for 2,4-DNP and plotting the ratios in a graph. With more conversion product accumulation and reduction in the concentration of 2,4-DNP, the ratio will move more toward 0 from 1. i.e., 1 will be the ratio for 2,4-DNP, which acts as the negative control in the experiment, and values will move toward 0 with the reduction in the concentration of 2,4-DNP in the supernatant.

### LC-MS

The bacterial cultures were grown in 5 ml MM with and without 2,4-DNP for 24 h at 37°C at 160 rpm. The cells were pelleted down at 10,000 × *g* for 10 mins, and the supernatant was collected in a fresh Falcon tube. The supernatant was filtered using a sterile syringe filter (Becton Dickinson) with a PVDF filter membrane of 0.22 µm pore size (GE Health Care UK Limited). LC-MS was performed in the negative ionization mode in LCESI Q Tof (Bruker Daltonics, Billerica, Massachusetts, USA). The compound spectra were analyzed using Compass Bruker Data Analysis software, and fragments were analyzed using the web tool Met-Frag. EGG was the database used during the fragment search. The spectrum was replotted using the software Origin-2018, and Met-Frag results were redrawn using the software ChemDraw.

### RNA purification, cDNA synthesis, and qRT-PCR

Total RNA was extracted from the WT and Δ*lon* grown for 3 h, then treated with 0.5 mM 2,4-DNP, and allowed to grow for 1 h and 3 h at 37°C and 160 rpm. Briefly, the cells were treated with TRIzol reagent (Sigma, St. Louis, Missouri, USA) for 1 h at 37°C in a shaker dry bath and lysed. The debris was pelleted down; the supernatant was transferred to fresh tubes and treated with chloroform (Sigma), and the organic phase was removed. The aqueous phase collected was treated with propanol (Sigma) to precipitate the RNA. The RNA was pelleted, washed with 70% chilled ethanol, dried, and dissolved in 15 µL DEPC-treated water. RNA concentration and purity were quantified using a NanoDrop spectrophotometer (Thermo Scientific, Waltham, MA, USA), followed by DNase treatment. Subsequently, the RNA was reverse-transcribed to cDNA using RevertAid (Thermo Scientific, Waltham, MA, USA). DNA contamination was tested by PCR amplification of *rrsC*. qPCR was carried out using the Bio-Rad CFX Connect System (Bio-Rad, CA, USA). The primers used are listed in Table S1. GapA was used as the reference gene for all conditions, and fold change was calculated using the 2^-∆∆Ct^ method. The primer efficiency for each primer pair was determined before performing PCRs, according to the MIQE guidelines (44).

### Cloning of *marA* for *trans* complementation

The calcium chloride method was used to prepare competent cells. Bacterial cultures were grown overnight from glycerol stock in the presence of antibiotics specific to the resistance cassette for the pre-inoculum. The pre-inoculum was normalized to O.D. 2, and 0.2% of O.D. 2 cells were cultured in 50 mL flasks till the mid-log phase (O.D. 0.4–O.D. 0.5). The flask was incubated at 4°C for 1.5 hours. Fifteen milliliters of the cell culture was pelleted down at 3,000 × *g* for 15 mins at 4°C. The cells were resuspended in 7.5 mL of filter-sterilized 100 mM MgCl_2_ (HiMedia), incubated at 4°C for 20 mins, and then pelleted down at 3,000 × *g* for 15 mins at 4°C. Following this, the cells were resuspended in 7.5 mL of filter-sterilized 100 mM CaCl_2_ (HiMedia), incubated at 4°C for 20 mins, and then pelleted down at 3 000 × *g* for 15 mins at 4°C. Then, the cells were gently resuspended in 1 mL of 100 mM CaCl_2_ in 15% glycerol. Aliquots of 100 μL were transferred to Eppendorf tubes, flash-frozen with liquid nitrogen, and stored at −80°C.

The WT MG1655 was used as the template for amplifying the *marA* gene using specific primers (Table S2) and Taq DNA polymerase (G-biosciences, Delhi, India). *marA* was targeted for cloning between EcoRI and HindIII sites in pQE60 plasmid (45, 46) using T4 DNA ligase (DX/DT, Bengaluru, India). The ligated pQE60 plasmid with the *marA* gene was amplified using the positive clones. The heat-shock method transformed the positive clones and control vectors into WT and Δ*marA*.

### Determination of minimum inhibitory concentration (MIC)

MIC for the bacterial strains was determined using the broth dilution method. The bacterial cultures grown overnight at 37°C and 160 rpm served as the pre-inoculum. Two-hundred microliters of the LB was aliquoted in a 96-well microtiter plate (Tarsons, Kolkata, India), followed by the addition of different concentrations of ciprofloxacin (HiMedia), ranging from 10 ng/mL to 100 ng/mL and tetracycline (HiMedia), ranging from 0.1 μg/mL to 0.9 μg/mL and ampicillin (HiMedia), ranging from 0.5 μg/mL to 5 μg/mL. All the wells used 0.2% of the OD2 culture as an inoculum. The plate was then incubated at 37℃ for 18 h, followed by measurement of O.D. values at 600 nm. The concentration of the antibiotic is reduced to half and is recorded as the MIC value.

### Statistical analysis

All statistical analyses were performed using ANOVA in GraphPad Prism 8, wherever applicable. Data were represented as mean ± S.D., where * denotes *P* ≤ 0.05, ** denotes *P* ≤ 0.01, *** denotes *P* ≤ 0.001, and **** denotes *P* ≤ 0.0001.

## SUPPLEMENTAL MATERIAL

Supplemental material aem.01603-25-s0001.pdf Tables S1 and S2; Fig. S1 to S5.

ASM does not own the copyrights to Supplemental Material that may be linked to, or accessed through, an article. The authors have granted ASM a non-exclusive, world-wide license to publish the Supplemental Material files. Please contact the corresponding author directly for reuse.
