# Supplementary material for: Involvement of Escherichia coli-encoded Lon protease and its substrates in phenotypic antibiotic resistance elicited by 4-amino-2-nitrophenol
Source: Appl Environ Microbiol. 2025 Dec 9;92(1):e01603-25. doi: 10.1128/aem.01603-25 (PMC12838266; doi:10.1128/aem.01603-25)
Supplement: Supplemental material — Tables S1 and S2; Fig. S1 to S5. [file aem.01603-25-s0001.pdf]

SUPPLEMENTARY INFORMATION

SI Table 1: List of primers used in the study

**Sl.No**	**Primer name**	**Sequence (5'-3')**
1	qRT *gapA* FP	TTTCCGTGCTGCTCAGAAAC
2	qRT *gapA* RP	GTCAACACCAACTTCGTCCC
3	qRT *nfsA* FP	GAACTTATTTGTGGCCATCG
4	qRT *nfsA* RP	TCACCAGTTCTTCACGTAAC
5	qRT *nfsB* FP	GGTTTATCTCAACGTCGGTA
6	qRT *nfsB* RP	GGTGTAGCCTTTCTCTTTCA
7	qRT*marA* FP	TGTCCAGGACGCAATACTGACG
8	qRT *marA* RP	TTTTGAAGGTTCGGGTCAGA
9	Comp *marA* FP	CCGGAATTCATGTCCAGACGCAATACTGACG
10	Comp *marA* RP	CCCAAGCTTGCGCGCCTAGCTGTTGTAATGATTTAAT

SI Table 2: List of plasmids used in the study

Sl No	Plasmid	Description	Reference
1	pQE60	Low copy bacterial expression plasmid used for trans complementation from the constitutive T5 promoter. It contains an Ampicillin resistance cassette	Chandra *et al.*,2017;2020
2	pBAD33	Low copy number expression vector regulated by the arabinose operon	Matange,2020
3	pBAD33-Lon	Plasmid for expression of Lon protease from an arabinose inducible promoter (Matange,2020)	Matange,2020

Supplementary Figures

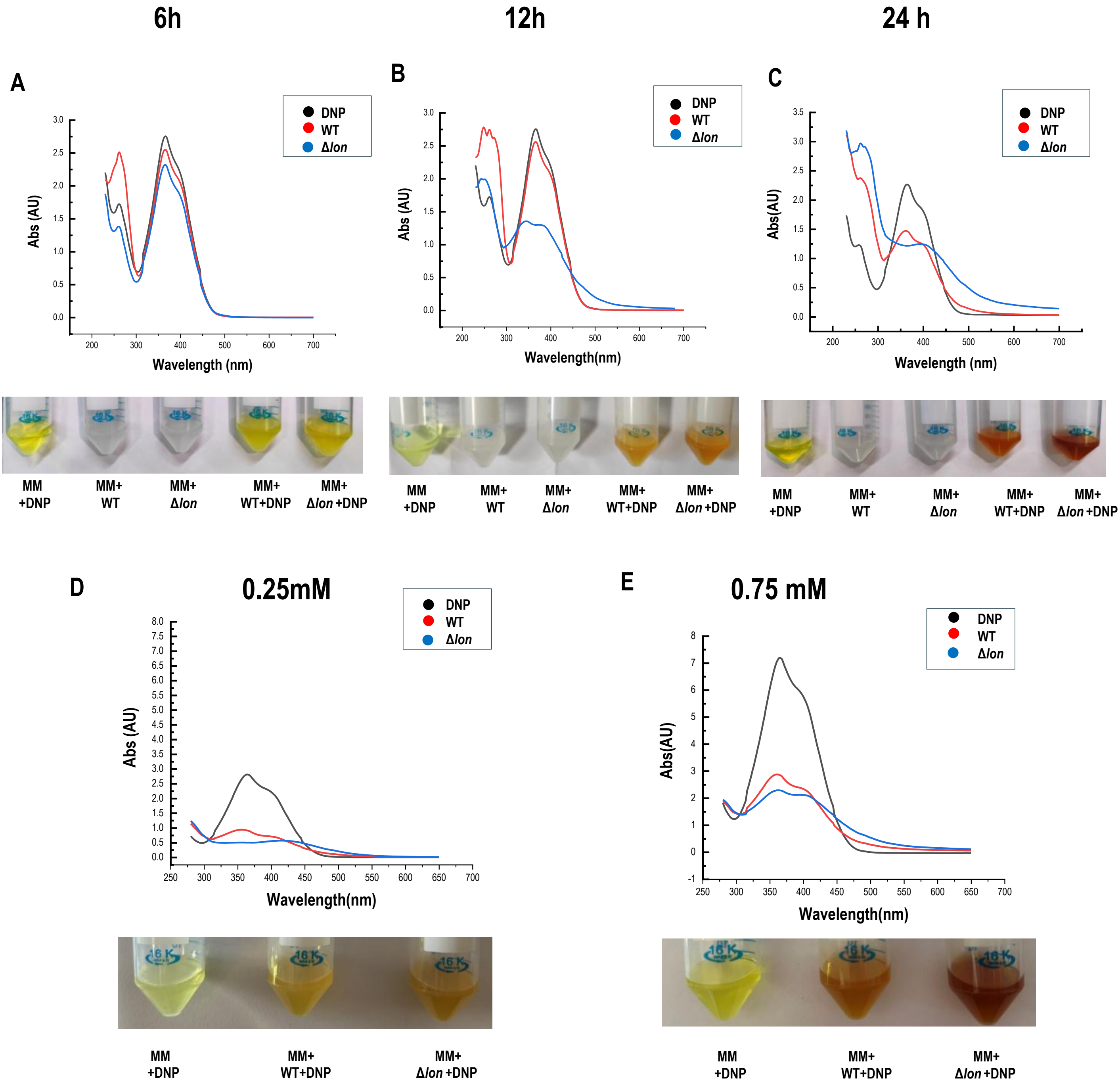

SI Fig1. A reddish brown-coloured compound is produced in higher amounts in the Δ*lon* cells grown in minimal media in the presence of 2,4-DNP . *E. coli* MG1655 WT and Δ*lon* strains were cultured for 6,12, and 24 h at 37 °C and 160 rpm in the presence of 0.5mM of 2,4-DNP. Culture supernatants were collected from bacterial culture grown in the absence and presence of 0.5 mM 2,4-DNP. Subsequently, the UV-visible spectrum of the supernatants was measured after diluting with minimal media: A) post 6 h of treatment; B) post 12 h of treatment; C) post 24 h of treatment. Culture supernatants were collected from bacterial cultures grown in the presence of (D) 0.25 mM and (E) 0.75 mM 2,4-DNP. Subsequently, the UV-visible spectrum of the supernatants was measured after diluting with minimal media

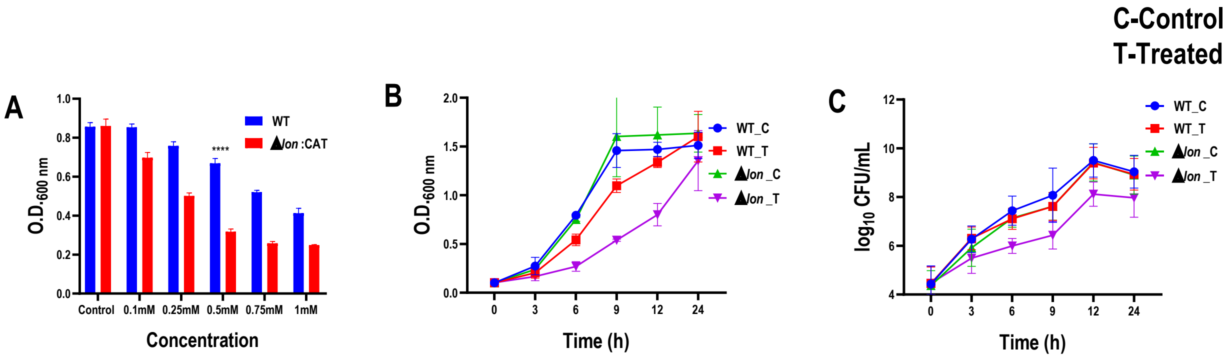

SI Fig 2. The higher conversion product seen in the *lon* mutant is not due to higher growth in the presence of 2,4-DNP. *E. coli* MG1655 WT and Δ*lon* strains were cultured for 6 h at 37°C and 160 rpm in the presence of different concentrations of 2, 4-DNP. (A) Growth was assayed by measuring the O.D at 600 nm using a UV-visible spectrophotometer. B) Bacterial growth curve plotted as OD/ml v/s time and C) Bacterial growth curve plotted as log CFU/ml v/s time showing growth reduction in Δ*lon* strain in the presence of 0.5mM 2,4-DNP compared to WT. C represents the control group and T represents the treated group. The data are representative of three independent experiments. For statistical analysis, two-way ANOVA was performed, and the data were plotted as mean ± S.D where * indicates $p<0.05$. Statistical analysis was performed between WT and Δ*lon* strains for the different conditions.

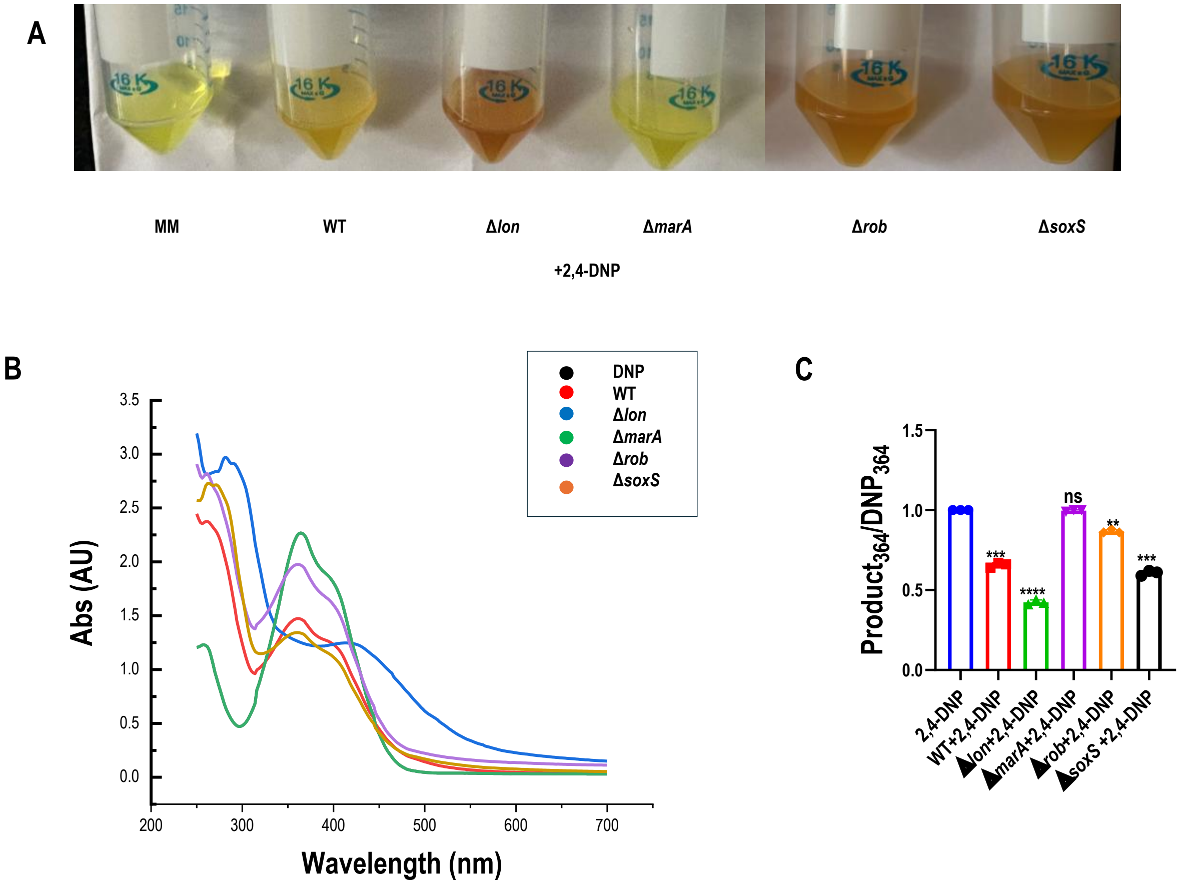

SI Fig 3: *rob* and *soxS* mutants convert 2,4-DNP to the reddish brown-coloured compound. *E. coli* WT, Δ*lon*, Δ*rob* and Δ*soxS* were cultured in the presence of 0.5mM 2, 4-DNP for a period of 18 h at 37^{0}C and 160 rpm. (A)Tubes showing conversion product formed post 18hr of treatment;(B) UV-visible spectrum of the supernatant after diluting 1:2; (C) graph showing quantification of spectrum. The data are representative of three independent experiments plotted as mean ± SD. * indicates P<0.05. Statistical analysis was performed for each strain relative to its untreated control. Comparison between the strains is indicated wherever significant.

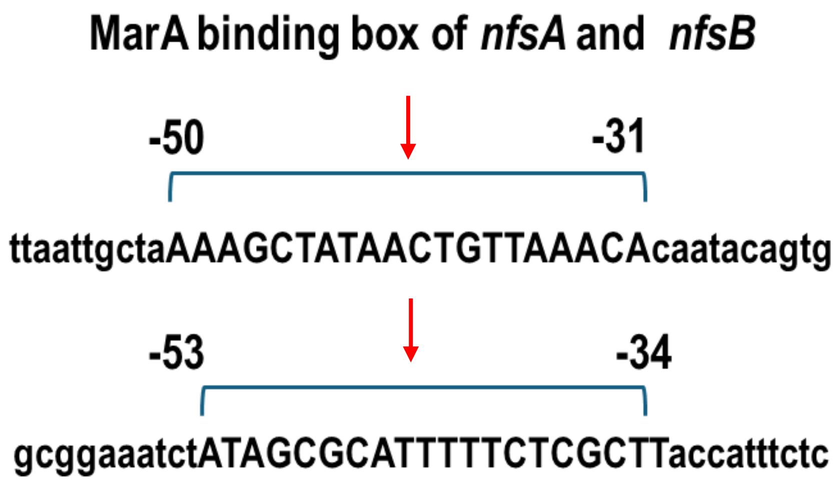

SI Fig 4 : Representative image of Mar A binding box of *nfsA* and *nfsB* : Position of Site Center Relative to Transcription Start Site for *nfsA* and *nfsB* (bp) are -40.5 and -43.5 respectively (marked with red arrow)

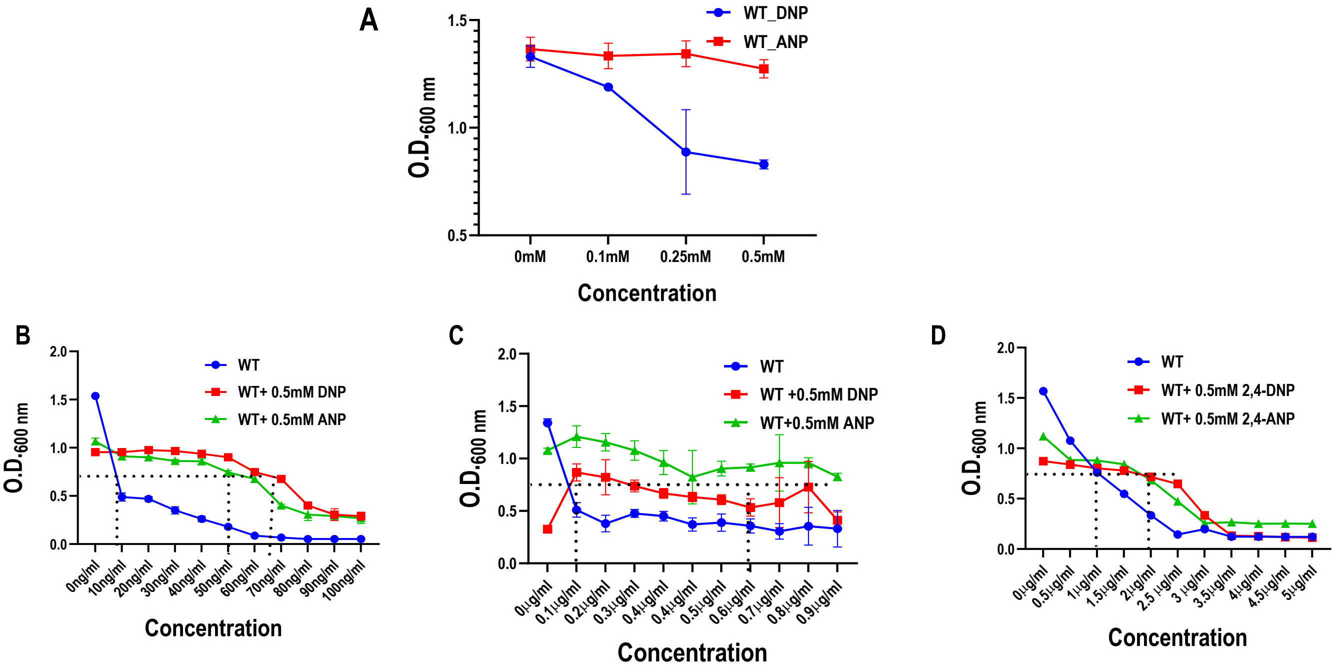

SI Fig 5. 4,2-ANP does not induce growth reduction in the WT strain. *E. coli* WT strain was cultured with concentrations of 2, 4-ANP and 4-DNP for 6 h at 37˚C and 160 rpm in LB. (A) The growth of WT strain under different concentrations of 2,4-DNP and 4,2-ANP. MIC broth dilution assay of WT with (B) ciprofloxacin, (C) Tetracycline and (D) ampicillin in the presence and absence of 0.5mM 2,4-DNP and 0.5mM 4,2-ANP was performed. The data are representative of at least three independent experiments plotted as mean ± SD.
